# A Fluorescent Biosensors for Detection Vital Body Fluids’ Agents

**DOI:** 10.3390/s18082357

**Published:** 2018-07-24

**Authors:** Witold Nawrot, Kamila Drzozga, Sylwia Baluta, Joanna Cabaj, Karol Malecha

**Affiliations:** 1 Faculty of Microsystem Electronics and Photonics, Wrocław University of Science and Technology, Wybrzeże Wyspiańskiego 27, 50-370 Wrocław, Poland; witold.nawrot@pwr.edu.pl; 2 Faculty of Chemistry, Wrocław University of Science and Technology, Wybrzeże Wyspiańskiego 27, 50-370 Wrocław, Poland; kamila.drzozga@pwr.edu.pl (K.D.); sylwia.baluta@pwr.edu.pl (S.B.); joanna.cabaj@pwr.edu.pl (J.C.)

**Keywords:** sensors, biosensors, fluorescence, biological samples, LTCC technology

## Abstract

The clinical applications of sensing tools (i.e., biosensors) for the monitoring of physiologically important analytes are very common. Nowadays, the biosensors are being increasingly used to detect physiologically important analytes in real biological samples (i.e., blood, plasma, urine, and saliva). This review focuses on biosensors that can be applied to continuous, time-resolved measurements with fluorescence. The material presents the fluorescent biosensors for the detection of neurotransmitters, hormones, and other human metabolites as glucose, lactate or uric acid. The construction of microfluidic devices based on fluorescence uses a variety of materials, fluorescent dyes, types of detectors, excitation sources, optical filters, and geometrical systems. Due to their small size, these devices can perform a full analysis. Microfluidics-based technologies have shown promising applications in several of the main laboratory techniques, including blood chemistries, immunoassays, nucleic-acid amplification tests. Of the all technologies that are used to manufacture microfluidic systems, the LTCC technique seems to be an interesting alternative. It allows easy integration of electronic and microfluidic components on a single ceramic substrate. Moreover, the LTCC material is biologically and chemically inert, and is resistant to high temperature and pressure. The combination of all these features makes the LTCC technology particularly useful for implementation of fluorescence-based detection in the ceramic microfluidic systems.

## 1. Introduction

Biosensors are analytical tools that measure the presence of a single molecular species in complex mixtures by combining the exquisite molecular recognition properties of biological macromolecules with signal transduction mechanisms that couple ligand binding to readily detectable physical changes [1]. These analytical systems have been extensively studied over the years due to their role in monitoring different types of analytes for food safety, environmental monitoring, and clinical analysis. The analytical performance clearly determines the final prototypes of clinical sensors for commercial use (i.e., glucose biosensors). As biosensors are essential for clinical analysis, we present an update on previous literature reviews. Their analytical performance is compared, and their advantages and limitations described based on the transduction principle.

The choice of the substrates for the analytical applications depends on the transduction methods; i.e., fluorescence, chemiluminescence, or amperometry—and on the purpose of the analytical measurements [2]. Various fluorimetric assays and fluorescent biosensors based on biocatalysts’ activity have been reported using organic dyes, inorganic semiconductor quantum dots (QDs), and carbon quantum dots as fluorimetric indicators [3]. 

Advances in miniaturization, nanotechnology, and microfluidics, together with developments in cloud-connected point-of-care (POC) diagnostics technologies are pushing the frontiers of POC devices toward cost-reasonable, easy to use, and enhanced sensitivity molecular-level diagnostics. The merger of various bio-sensing systems within smartphone-integrated electronic readers provides proper on-site and real-time diagnostics based on various types of chemical and biological targets [4]. 

This review focuses on biosensors that can be applied to continuous, time-resolved measurements with fluorescence. The material presents the fluorescent biosensors for the detection of neurotransmitters, hormones, and other human metabolites as glucose, lactate, or uric acid.

## 2. Point-Of-Care—Vital Body Fluids’ Agents

One of the most dynamically developing areas of modern medicine is, according to the motto “prevention is better than cure”, clinical diagnostics. Researchers are focused not only on improving existing and constructing new analytical devices, but also more and more accurate methods of detecting chemical or biochemical compounds that show how the occurrence of given pathologies are being developed. During quick tests, so-called “bedside”, the time of the analysis is extremely important. Tests carried out in intensive care centers require the maximum time to wait for the results of the patient, which is often caused by the need to take immediate therapeutic decisions (sometimes access to the parameters obtained is necessary at the time). The term turnaround time (TAT) defines the time in which the sign should be made [5].

For the sake of many stages during the analysis (including patient preparation, material collection, transport, registration, and execution of the designation), the TAT behavior and the receipt of results in real time are not ensured. Hence, research is more often used as a point-of-care (POC), which means research in the place of patient care or bedside laboratory tests. 

The beginning of the use of diagnostic systems for POC research is considered to be the 60’s and 70’s of the 20th century, which is the date of development and implementation of a glucometer, an all-in-one apparatus enabling independent measurement of glucose in blood. Towards the end of the 1970s, one-time tests, such as pregnancy tests, have revolutionized the testing of disposable household products. Currently, we can find fertility tests, menopausal tests, tests detecting the presence of psychoactive substances, or the presence of infection. Designing devices allowing POC analysis in the clinical sense (enabling continuous monitoring of given substances) has been developing rapidly for two decades, enabling quick and sensitive “bedside” diagnostics [6].

In cases of life-threatening situations, such as multi-organ injuries, extensive post-operative injuries, systemic inflammation, generalized infections (sepsis), heart failure, and respiratory failure, it is important to shorten the TAT as much as possible only with the POC test tools. It is also important that such clinical parameters, measured at the time of life threatening, are dynamic in the patient and often require immediate medical intervention [6].

The ideal point-of-care testing (POCT) device should be characterized by small size, resistance to any interferences, required low sample volume, and a linear response to the determined concentrations of the analyte to be tested. The disposable parts of such a device must be cheap and easy to use. Of course, these requirements are contradictory and make the construction of such a POCT device quite a challenge [7].

The use of detection mechanisms associated with fluorescence changes in the microfluidic channels leads to the creation of highly integrated systems requiring low financial outlay, and at the same time enabling determination of concentration in the nano/pikomolar range. In recent years, many research groups have used laser-induced fluorescence in microfluidic chips, such as lab-on-chip microsystems [8], capillary electrophoresis [9,10,11] or miniature biosensor devices [12,13,14]. Depending on the purpose and implementation of the tests, the determination is carried out in the volume of liquid (e.g., capillary electrophoresis) or on a functionalized surface [15,16]. One of the most frequently used mechanisms is Total Internal Reflection Fluorescence (TIRF), which stimulates fluorescently determined analytes on the surface of the flow channel using a evanescent waveguide [17,18,19,20].

The construction of microfluidic devices based on fluorescence phenomena uses a variety of materials, fluorescent dyes, types of detectors, excitation sources, optical filters, and geometrical systems. Therefore, there is an unlimited number of possibilities to combine them. Designing POTC devices is associated with the use problem, which largely depends on the strategy of replacing one-off parts. Extensive settlement procedures are very often time-consuming and require the recruitment of specially trained personnel. Thus, the conversion concept and the design of microfluidic chips are the key factors for the practical application of the device [21].

The solution to these problems are biosensor devices, which in combination with microfluidic systems offer an integrated and miniaturized alternative to traditional, repeatable laboratory methods. Due to their small size, these devices can perform a full analysis, including sampling, separation, mixing and determination of concentration with a simultaneous reduction of costs and an increase in the limits of specificity and sensitivity of detection in comparison to the usual analytical methods. What is more, microfluidic biosensors, thanks to high throughput, real-time work and fast reaction, are becoming an ideal tool in POC analysis [22,23].

## 3. Fluorescent Biosensors for Determination of Hormones

### 3.1. Characteristic of the Exemplary Hormones

Hormones play an extremely important role in the body, acting as regulatory molecules, integrating the most important living systems. Unfortunately, sometimes their presence or too high concentration may lead to serious disorders, environmental pollution or they may be used as doping substances.

Endocrine disrupting compounds (EDCs) are a group of specific exogenous contaminants, which includes polychlorinated dibenzodioxins (PDCC), polychlorinated biphenyls (PCB), phthalates, alkylphenols, and active substances contained in pesticides (biocides) [24]. In addition to these compounds, natural hormones as well as synthetic hormones that are components of pharmaceuticals are also mentioned among EDCs. EDCs are substances that cause disturbances in the production, release, transport, action, binding, and metabolism of endogenous hormones and their receptors in the body [25]. To these compounds belong estrogens derivatives, such as: estrone (E1), estradiol (E2), estriol (E3), and estetrol (E4).

The action of endocrine active compounds is long-lasting, but the basic difficulty in preventing adverse effects on organisms is that they do not initially cause symptoms. These substances accumulate in the body and their effect may occur after several dozen years of exposure. As has been written, hormones behave similar to natural and synthetic estrogens. The action of hormones is primarily based on the total or partial imitation of steroid hormones by interacting with hormonal receptors or affecting the signaling pathways between cells. They can also block or hinder the formation of normal signaling compounds with their target receptors. Anti-estrogens or anti-androgens have such properties. Hormones may also affect the production or distribution of hormones normally present in organisms and affect the malfunctioning of hormone receptors [26,27].

Anabolic androgens steroids (AAS) is the group of most frequently detected doping substances in sports [28]. Mostly, they are used by athletes to increase muscle mass and efficiency of the organism. World Anti-Doping Agency (WADA) have prohibited the use of AAS because of the negative impact on the body and to ensure fair-play [29]. These compounds are derivatives of testosterone and 19-nortestosterone, and their action is based on the binding and activation of androgen receptors, which are involved in the synthesis of proteins and calcium [30]. Testosterone (17β-hydroxyandrost-4-en-3-one or α-4-androsten-17β-ol-3-one) playing important role in male sexual differentiation, synthesis of protein and human physical performance [31].

Insulin is one of the most important anabolic hormones in the human body. It is a peptide hormone, produced by beta cells of the pancreatic islets [32]. Disorders in the amount of glucose caused by improper insulin action are the cause of severe illnesses and dysfunctions in humans, which are called diabetes mellitus [33]. Fast and sensitive detection of this hormone is very important not only because of the diagnostic of diabetes but also because of the improper use of insulin as a doping drug in competitive sports [34].

### 3.2. Detection Strategies of Working Fluorescent Biosensor for Hormones Monitoring

The increasing number of pollutants resulting from the presence of EDC, the use of hormones as doping agents, and a number of diseases resulting from improper functioning of the endocrine system requires the construction of analytical devices that are precise, sensitive, specific, and easily enable quick and easy detection of these substances in target fluids. Optical biosensors are becoming a powerful alternative to current analytical techniques, enabling rapid monitoring of these compounds in real time.

**Figure 1:**
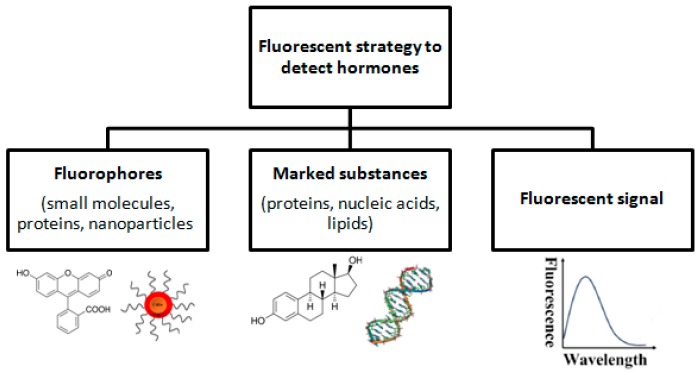
Fluorescent strategy to detect hormones.

Fluorescence is the result of a three-stage process (excitation, life time in fluorescence, and fluorescence emission) that occurs in some molecules called fluorophores or fluorescent dyes. The fluorophore molecules can be small molecules, proteins or nanoparticles (e.g., quantum dots) that can be used to label proteins, nucleic acids or lipids (Figure 1) [35]. The use of the appropriate length of light may cause excitation of the fluorophore molecule, which may result in an increase in fluorescence intensity or its decreasing (e.g., due to the FRET phenomenon). Fluorescence detection often includes: a source of excitation light (e.g., LED photodiodes), a fluorophore particle, wavelength filters to isolate photon emissions from excitable molecules, and a detector that records changes in fluorescence intensity and generates a measurable value [35].

The fluorescence strategy is often used in sensing systems, for example Weina Ming et al. developed a fluorescent method for the detection of 17β-estradiol in aqueous solutions. They used the phenomenon of competitive hormone adsorption with fluorescein, which in the proposed system serves as a fluorophore particle. Functionalization was performed on a new magnetic core-shell material that combines the surface application technique and the ability to separate magnetic Fe_3_O_4_ nanoparticles. Fe_3_O_4_ nanoparticles have been modified with acrylic acid (AA) and fluorescein. 17β-estradiol was added to the thus-modified particles due to its complementary affinity for fluorescein. The change in fluorescence intensity indicated the 17β-estradiol concentration in the test solution [36].

### 3.3. Fluorescent Biosensors for Detection of Hormones—Overview

A fluorescent-based biosensor for the detection of 17β-estradiol was constructed by Yildirim et al. using specific short chain oligonucleotides (aptamers) [37]. For this purpose, 17β-estradiol specific DNA aptamers were used. The analytes were fluorescence labeled and excited with a laser beam at 635 nm. The mixture was pumped to the photosensitive unit and the remaining free aptamers bound to the estradiol immobilized on the surface of the sensor and gave a fluorescence signal, which is the target concentration of estradiol. The sensing process can be complete in less than 10 min, with a detection limit of 2.1 nM.

A gold nanoparticles-based fluorescence immunoassay was presented [38] to ultrasensitive detection of 17β-estradiol. The sensing system consist two types of nanoparticles: magnetic microparticles (MMPs), which were functionalized with and anti-17β-estradiol antibody produced in rabbit as a capture probe and double codified gold nanoparticles modified with biotin and anti-rabbit antibody as a signal amplifier. Under optimized conditions the detection limit was found as 6.37 × 10^−6^ ng mL^−1^. The constructed biosensor was used for the detection and determination of 17β-estradiol in human urine.

A TIRF-based biosensor for detection of steroid hormone testosterone was also constructed [39]. For this purpose, monoclonal antibodies against testosterone were used. The constructed biosensor was used to determine a testosterone in lab water, drinking water and river water with a detection limit of 0.2 ng L^−1^.

Biosensors for detecting and determining insulin have also been constructed [34,40]. Yuhui Wang with co-workers have designed an optical biosensor based on the FRET phenomenon. The detection system included low-infrared quantum dots (NIR-QDs) covalently modified with insulin aptamers serving as an energy donor and imprinted carbon nanoparticles as an energy acceptor. The constructed system was used to detect insulin with a detection limit of 0.72 pM [40]. Han Zhang et al. proposed a sensor system based on energy transfer from PFO (donor) to PTCA (acceptor). In the constructed PFO system, they were combined with the primary insulin antibody, which can then interact with insulin through specific recognition of the antibody and antigen. Under the optimal experimental conditions, the constructed biosensor exhibited a low detection limit of 3.0 × 10^−6^ ng mol^−1^ [34].

Table 1 presents exemplary optical biosensors and sensors for hormones detection.

## 4. Fluorescent Biosensors for Determination of Neurotransmitters

Neurochemical methods are generally used for mechanism investigations of brain action, understanding the presence of neurotoxins during some disorders, and also for understanding the relationship between chemistry in central nervous system (CNS) and the emotional state [42,43]. The proper brain and CNS action is strictly connected with the proper level of the neurotransmitters and/or metabolism of these molecules. Neurotransmitters act as neurochemical messengers, so any problems with regulation in neurotransmission may cause a wide range of ailments, starting from the neurological as Parkinson’s disease [44,45], Alzheimer disease [46], Huntington’s disease [47], to psychiatric conditions such as schizophrenia [48], depression [49], or even cardiovascular and digestive complaints [50,51]. As neurotransmitter may act as such molecules: amino acids (e.g., glutamate, aspartic acid), derivative of amines (dopamine, serotonin), small peptides (such as galanin or angiotensin), nucleosides (ATP, adenosine), and acetylocholine. Sensing the neurotransmitters in vivo is fraught with a lot of difficulties and challenges associated mostly with the rapid and heterogeneous changes that occur in the CNS. The analytical measurements of described biomolecules may be provided by combining gas chromatography with mass spectroscopy (GC-MS) [52], high performance liquid chromatography (HPLC) [53], capillary electrophoresis (CE) [54], electrochemistry methods such as cyclic voltammetry analysis [55] or differential pulse voltammetry [56], chemiluminescence [57] or spectrophotometry [58]. However, most of these methods do not permit high sensitivity, selectivity, quickness, and do not allow for miniaturization and automatization in the analysis. Due to this, the promising measurement technique is biosensors, which can be directly used, possess a possibility for the automatization of the survey, allow for the monitoring more than one analyte at a time and are highly selective, according to the presence of biologically active material. 

Determining the level of neurochemicals in body fluids is essential for the industry. For instance in environmental monitoring, food analysis or clinical diagnostics, is currently not available. This chapter presents new optical methods for neurotransmitter determination, which also in the future will be capable of monitoring these compounds in body fluids.

### 4.1. Characteristic of the Exemplary Neurotransmitters

The main mediators of the autonomic nervous system, which have a stimulating effect on the sympathetic nervous system, are: dopamine, epinephrine (adrenaline) and norepinephrine (noradrenaline), which belongs to the catecholamines, and serotonin (5HT) (monoamine). Monitoring and detection of these molecules is extremely important in modern medicine, specifically for helping point-of-care measurements in hospitals [59,60].

Generally, the endogenous catecholamine concentration, which has been measured for patients without any diagnosed disorders, was approximately 150–800 ng/L for NE and 10–50 ng/L for EP and dopamine (DA) [21]. Their level can be measured in different biological fluids, such as plasma, saliva, urine, or blood serum [61].

DA plays a significant role in movement, muscle tension control, pain processing, arousal, motivation, and reward [62]. As a result of its importance as a neurotransmitter, deficiency or excessive secretion of DA may cause serious diseases, like psychosis, depression, attention deficit hyperactivity disorder (ADHD), and Parkinson’s syndrome [63]. Epinephrine (EP) plays a key role in the stress mechanism—the rapid reaction of mammals to a threat. In addition, EP regulates the level of glucose in the blood by increasing breakdown of glycogen to glucose in the liver. Due to this fact, any changes in the level of EP in the human body may cause disorders connected with the central nervous or cardiovascular systems (e.g., heart attack) [51]. Norepinephrine (NE) has a similar action to EP. In the brain, NE is responsible for increasing arousal and alertness, supports wakefulness, enhances memory and recalling, and enables concentration. In the rest of the body this catecholamine increases the heartbeat and blood pressure, releases stored glucose, accelerates blood flow to the skeletal muscles, reduces blood flow to the digestive system, and inhibits bladder emptying and motor activity in the gastrointestinal tract [64].

### 4.2. Detection Strategies of Working Fluorescent Biosensor for Catecholamines Monitoring

**Figure 2:**
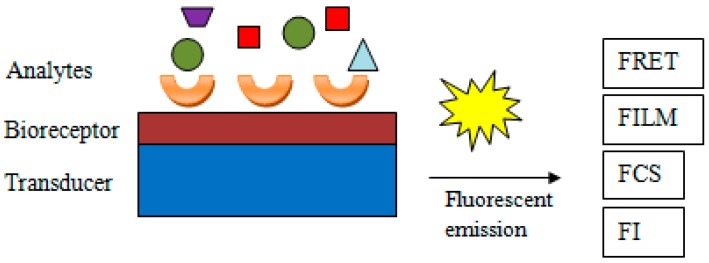
Methods for fluorescence determination using biosensors (general scheme).

To determine the catecholamines using an optical detection bio-system, fluorescence-based biosensors are generally used (Figure 2). Fluorescent biosensors are analytical devices for the noninvasive detection of biomolecules present in biological samples, and their work is based on the fluorescence phenomenon that occurs when electromagnetic radiation is absorbed by fluorophores or fluorescently labeled molecules [46]. The created signal is mostly determined by employing one of the following techniques:

- FRET (Förster Resonance Energy Transfer),
- FLIM (Fluorescence Lifetime Imaging),
- FCS (Fluorescence Correlation Spectroscopy),
- FI (changes in Fluorescence Intensity).

FRET is a method involving the radiation-less transfer of energy from a “donor” fluorophore to an “acceptor” fluorophore [65]. Such a phenomenon occurs when the distance between the donor and acceptor is not larger than 10 nm and the dipoles of both molecules are oriented suitably. The determination is based on quenching the fluorescence in most cases. 

FLIM is a technique used for imaging processes which take place in living cells. Such an occurrence enables an average lifetime when a molecule remains in its excited state after absorbing a photon to be detected [66]. Such a method is used to gather data about changes in the fluorophore local environment or for changes in its energy [67].

FCS is based on small deviations in an unprompted fluorescence intensity of the sample and the obtained signal provides information about the kinetics of thermodynamic processes related to reversible fluorescence changes [66,67].

FI is a method based on the direct measurement of fluorescence intensity (fluorescence emission) to the response of the system to excitation. FI is generally employed for fluorescence investigations of biosensors containing an enzyme in the bioreceptor part [68,69].

### 4.3. Fluorescent Biosensors for Detection of Catecholamines—Overview

At present, there is no available diagnostic tool for the sensitive determination of catecholamines, which is extremely important in modern medicine. Optical bio-systems are a very promising candidate for analytical sensors, because they provide fast, simple, cheap, and sensitive analysis of the investigated sample. 

In the literature are presented fluorescent detection methods for catecholamines investigations, however, most of them are connected with DA detection [70,71]. In the case of EP and NE, there are almost no existing detection systems, which is opposite to DA [72,73]. It is much more difficult to detect EP or NE because of their rapid metabolism and in fact, these molecules do not possess any fluorescence properties [74].

**Figure 3:**
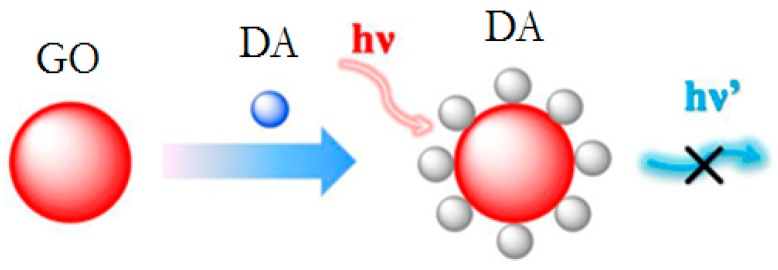
A general scheme for quenching the high fluorescence of nanoparticles by dopamine (DA).

Baron et al. proposed a detection method for neurotransmitters using tyrosinase in a bioreceptor layer: dopamine, L-DOPA, epinephrine, and norepinephrine, which mediate the generation and growth of Au nanoparticles (Au-NPs). The assumption of measurements was based on the plasmon absorbance of the Au-NPs, which allows for the quantitative colorimetric detection of the neurotransmitters. Authors obtained good results of the detection limit (LOD; 2.5 µM for DA, L-DOPA and NE; 20 µM for EP) for a linear response in the range of 40–200 µM [75]. Nikolelis with a group presented a technique using a simple and sensitive spot test for the rapid one-shot detection of dopamine in human urine using lipid films with an incorporated resorcin derivative receptor. The lipid films without the receptor provided fluorescence under a UV lamp, while the use of the receptor in these films quenched the fluorescence. A drop of dopamine or urine containing such a molecule provided a “switching on” of the fluorescence, which allows the determination at 10^−8^ M concentrations [76]. Chen et al. reported a graphene oxide-based (GO) photoinduced charge transfer (PCT) label-free near-infrared (near-IR) fluorescent biosensor for DA determination. Measurements based on the multiple noncovalent interactions between GO and DA resulted in effective self-assembly of DA on the surface of GO, and significant fluorescence quenching (general scheme of quenching presented on Figure 3). The developed method showed a detection limit equal to 94 nM [77].

Lin et al. introduced the DNA-biosensor for DA detection, based on a DNA-mediated silver nanostructure, which consisted of a large fluorescence enhancement resulting from specific binding of intercalating dye with the DNA that is released by DA from the nanoparticles. Such a prepared detection system was linear in a wide range of concentrations, from 0–200 nM, and showed great a detection limit of 6 nM [78]. Aswathy and Sony revealed a fluorescence-based sensor for DA determination. The analysis was based on the fluorescence quenching of the complex BSA stabilized gold nanoclusters (BSA–Au NCs) by the Cu^2+^ ions, and then the addition of a DA molecule, which causes the retrieval fluorescence by binding with the metal ion and removing it from the surface of BSA–Au NCs. The detection limit for such an arrangement equals to 0.01 µM and the linear response of DA in the range of 0.5–4 µM [79].

There has been also presented the microfluidic ceramic settings for dopamine detection based on polydopamine-graphene quantum dots with LOD 80 nM [80].

Table 2 presents aexemplary optical biosensors and sensors for neurotransmitters detection.

Searching for new approaches for neurotransmitter determination is a key factor in improving clinical diagnostics, which is directly connected with the point-of-care measurements. Such analytical tools will, in the future, ensure fast, simple, and bedside analysis of important biomolecules.

## 5. Fluorescent Biosensors for Determination of Key Metabolites

### 5.1. Characteristic of the Exemplary Key Metabolites

Highly selective and sensitive detection of biologically important structures, including small molecules (e.g., glucose, lactate, uric acid) and whole cells (e.g., pathogen microorganism and cancer cells), is critical in clinical analysis, environmental screening, food safety, and bioprocess monitoring.

Many studies in recent decades have focused on the development of sensor systems for the determination and monitoring of lactate concentration, which are a very important factor in clinical diagnostics, sports medicine, and food technology [86]. During the biochemical synthesis of lactates, the concentration of protons in cells increases. When the lactate synthesis rate is too high, the pH of the cells drops, which can lead to cellular acidosis, which pushes the muscles [87]. That’s why monitoring lactate is so important in the strength assessment and overall physical level of athletes [88]. 

Uric acid (UA, 2,6,8-trihydroxypurine) is the main end product of purine nucleotide metabolism, mainly found in urine and serum [89,90]. The UA concentration in a healthy person varies between 0.13 and 0.46 mM in plasma and between 1.49–4.46 mM in urine [91]. Disorders of uric acid levels are the cause of many diseases such as gout, hypertension, pneumonia, kidney damage, and a whole range of cardiovascular diseases. In addition, abnormal UA concentration can also cause central nervous system disorders, such as multiple sclerosis, Parkinson’s disease, or Alzheimer’s disease [92,93]. Due to the occurrence of numerous diseases associated with abnormal UA concentration, it is necessary to find fast and sensitive methods for determining this compound. For this purpose, various analytical techniques are used, such as spectrophotometry [94], electrochemistry [95], liquid chromatography [96] and methods using fluorescence [97]. Among the mentioned methods, fluorescent methods deserve special attention due to a number of benefits resulting from their use such as increased sensitivity, safety, simplicity, and speed of response.

Glucose is one of the most important molecules in the body. It performs the basic function in metabolic processes and is the main source of energy in cellular metabolism. As with other important molecules, glucose disturbances can cause many serious diseases such as diabetes or hypoglycemia. As the incidence of diabetes continues to increase, it is extremely important to monitor the level of glucose in the blood in order to accurately diagnose diabetes [98].

### 5.2. Detection Strategies of Working Fluorescent Biosensor for Metabolites Monitoring

**Figure 4:**
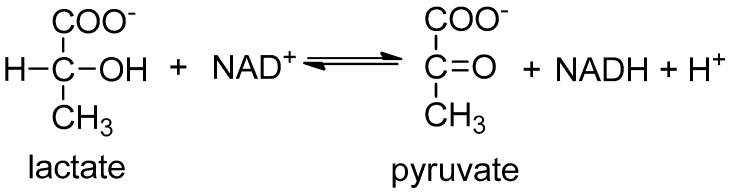
Fluorescent strategy to detect lactate with lactate dehydrogenase (LDH).

The most commonly used biologically active compound used to detect lactate is lactate dehydrogenase (LDH) (Figure 4). Sensors based on this enzyme utilize the fluorescent properties of the NADH coenzyme, which exhibits light absorption at 340–360 nm and is highly fluorescent in the 450–460 nm range. The intensity of fluorescence is directly proportional to the concentration of NADH, and thus to the amount of the substance being determined [86].

In the case of a fluorescent-based biosensor for glucose determination, the most widely used is glucose oxidase (GOx), which catalyzes the conversion of d-glucose and oxygen to d-glucono-1,5-lactone and hydrogen peroxide. 

One of the used strategies for fluorescence-based glucose sensing is the method based on the intrinsic fluorescence of the enzyme. Due to the presence of tyrosine and tryptophan in protein molecule, GOx exhibits a strong fluorescence signal with excitation at 224 and 278 nm, and emission at 334 nm [99]. A fluorescence indicator may be used as a GOx cofactor; FAD (flavin adenine dinucleotide) shows a fluorescence band at 520 nm, which is then quenched by the nearest amino acids. However, the intrinsic GOx fluorescence at 334 nm can be utilized for the glucose detection. During such an enzymatic reaction, any change in the fluorescence intensity of the protein is interdependent with the level of the glucose in the analyte [100].

**Figure 5:**
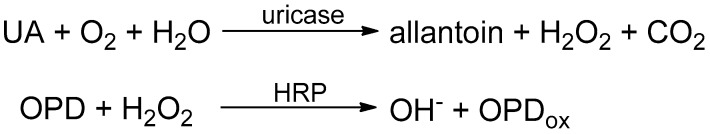
Fluorescent strategy to detect uric acid using lactate uricase and horseradish peroxidase (HRP).

Enzymatic systems can also be used to construct specific uric acid sensors. The detection process consists in carrying out a series of enzymatic reactions using uricase and horseradish peroxidase (HRP). The reaction consists in the initial oxidation of uric acid to allantoin and hydrogen peroxide (Figure 5). The resulting hydrogen peroxide can then oxidize o-phenylenediamine (OPD) in order to obtain a yellow color and an absorption peak at 450 nm [101].

### 5.3. Fluorescent Biosensors for Detection of Metabolites—Overview

Various biosensors based on fluorescence measurements for the determination of lactate have been developed. Yong-Sheng et al. used the enzyme lactate dehydrogenase (LDH) for the construction of a biosensor. LDH was immobilized in the inner surface in a medical capillary. The constructed biosensor can be applied for rapid and sensitive determination of lactate in ferment foodstuff, leechdom, and blood samples with LOD 0.45 mM [102]. Zheng et al. used an optical fiber and LDH enzyme for construction of the biosensor for determination of lactate in a single cancer cell [103]. For this purpose, they immobilized LDH on the nanosized tip of an optical fiber. Such a constructed biosensor was able to determine the concentration of lactate in single HeLa, MCF-7 and human fetal osteoblast cell with LOD 20 μM. 

Fluorescence strategies can be also used for the determination of uric acid. Azmi et al. designed an enzymatic biosensor to determine the concentration of uric acid in urine [91]. For the sensor construction, the combination of CDs quantum dots and two enzymes, uricase and horseradish peroxidase, were used. The hydrogen peroxide produced by the enzymatic reaction was able to quench the fluorescence of quantum dots, which was proportional to the concentration of uric acid in the test solution. The LOD of the proposed biosensor was found as 125 μM. Uricase was also used by the Long Q group [101]. The proposed method is based on the fact that uricase can oxidize uric acid to allantoin and hydrogen peroxide, which can oxidize o-phenylenediamine (OPD) to the oxidized OPD (oxOPD). The fluorescence of prepared UCNPs can be significantly quenched by oxOPD through inner filter effects (IFE).

Biosensors for the detection of glucose have also been constructed. Lin et al. proposed an enzyme based platform for the detection of this carbohydrate. The sensing system was constructed by using the label-free MIL-53(Fe) nanozyme and glucose oxidase [104]. Occurring enzymatic reactions cause changes in fluorescence intensity, which correspond to particular glucose concentrations. A biosensor constructed by this way can be certainly used to determine glucose concentration in normal and diabetic human serum samples with a detection limit of 8.44 × 10^−9^ mol L^−1^.

Chen et al. designed an enzymatic biosensor for glucose detection, based on the FRET phenomenon [105]. The CdSe/ZnS quantum dots were used as the donor, while the acceptor function was a green malachite binding dextran, which was conjugated with concanavalin A (Con A)—an enzyme with high fertility to glucose. In the presence of glucose, the CdSe/ZnS QDs emission quenched due to the FRET mechanism, is restored by replacing the dextran from Con A.

Ayranci et al. constructed another platform for measuring changes in fluorescence intensity, produced by the copolymerization of an electroactive rhodamine-based monomer (RDC) and a monomer containing a functional group (SNS) allowing binding of the enzyme [106]. The platform constructed in this way was used to measure the glucose concentration in the samples tested. The operation of the sensor results from the consumption of dissolved oxygen, which is a fluorescing quenching agent during the reaction of glucose oxidase with glucose. The detection limit for such a structure is 0.0012 mM.

Table 3 presents exemplary fluorescence-based biosensors for metabolites determination.

## 6. Technology of Microfluidic Systems with Fluorescence Detection

Several technologies are used for microfluidic system manufacturing, primarily: silicon, glass, polymer, and ceramic. The first can incorporate very fine structures by using various etching techniques (e.g., wet, DRIE). These processes are very well known and widely used, as they derivate from electronic component manufacturing methods. Therefore, highly integrated Micro Electro-Mechanical Systems (MEMS) are very common and cheap in mass production. However, development and small scale manufacturing are quite expensive due to high material and processing costs. The main issue in fluorescence measurement is material transparency. Therefore, silicon technology cannot be used as a standalone. In the case of silicon microsystems, bonding with glass is used in the vast majority of cases, as it is carried out using typical silicon bonding techniques, such as anodic bonding [107]. Full glass microsystems are rare, as it is a rather difficult material to process. Nonetheless, such technologies are being used, mainly due to exemplary optical properties and the chemical resistance of the material (borosilicate glass is the most common). Fabrication of microfluidic structures is carried out through a wet etching, milling, sanding, and laser techniques. In order to obtain closed canals, substrates need to be bonded either to another glass, silicon (through anodic bonding) [108], or polymer layer (trough plasma bonding) [109]. Therefore, implementation of complex spatial structures in glass microsystems is difficult. This technology is very suitable for manufacturing simple microfluidic fluorescence sensors, however, the excitation spectra must be significantly different than the emissions.

Polymer microsystems form a vast group. Nonetheless, the main trends can be separated: molding, 3D printing, and layer structuration. While the first is well established, the second is only just emerging, and the third is rather niche as a microfluidic technique. Molded structures are made primarily of optically transparent polydimethylsiloxane (PDMS). Forms can be made from various materials: from silicon (soft lithography) to 3D printed polymers. This method is inexpensive and relatively easy to develop. In order to obtain closed canals, the mold needs to be bonded, either to another PDMS structure or a different material. A plasma treatment is a common method. Due to good optical properties this material is very suitable for a fluorescence system manufacturing standalone [110] or in tandem with glass, as mentioned earlier.

Another technique, 3D printing of microfluidic structures is very promising. The most common and inexpensive method, Fused Deposition Modelling (FDM), also known as Fused Filament Fabrication (FFF), uses thermoplastic polymers, as in typical plastic processing, in the form of a cable that is heated and then pushed out through an extruder. This method, although very inexpensive in both equipment and materials, struggles with shape fidelity, reproducibility, leaking and transparency. The inkjet 3D printers are much more capable, although 10–100 times more expensive [111]. Foremost, they allow for fluorescence measurements [112,113]. Still, many polymers struggle with chemical resistance and biocompatibility. 

The layer structuration method is based on cutting or engraving patterns on subsequent polymer substrates using a cutting plotter, milling machine or laser system, and then laminating them at elevated temperature. Polymethylmethacrylate (PMMA) and polyvinylchloride (PVC) are most commonly used to produce microfluidic chips using this technique. Main issues of this method are poor canal quality, detail resolution and shape fidelity, as well as deterioration of transparency in case of laser engraving. 

Ceramic technologywas developed primarily in order to manufacture microelectronic devices. It is the least known, however, the immense capabilities of developing 3D structures in Low Temperature Cofired Ceramic (LTCC) proved to be very useful in microfluidics. The LTCC substrates are composed of ceramic particles and glass. It has the possibility of cofiring with thick film layers (e.g., Ag, Au, Pt, PdAg), which is useful not only for the integration of electronic components but also for the deposition or immobilization of functional materials. One of the first efforts to obtain a fluorescence measurement in a ceramic system was a sensor developed by Golonka et al. [114]. It consisted of three optical fibers inserted at an angle into microfluidic channels and glued in order to prevent leaking. The obtained measurements were noisy and inconsistent, probably due to the uncontrolled waveguide deflection. Moreover, the light intensity was very low due to the small optical fiber core diameter. Then Bembnowicz et al. made a microsystem for polymerase chain reaction (PCR) [115] with excitation through a planar waveguide made of a glass layer. One of main issues was the high firing temperature, which can cause the glass to soften and deform, resulting in incorrect light propagation and consequently insufficient excitation. It was overcome by integrating the waveguide after firing and then post-firing with a glaze layer in a lower temperature. Bembnowicz and Golonka made a breakthrough by developing a method for the integration of glass windows in LTCC microfluidic systems [116]. It relies on a suitable glass softening temperature: it has to be low enough so the window is flexible during ceramic shrinking but on the other hand the glass has to be sufficiently rigid to hold above voids. In the other case either the tension would be introduced or canals would be clogged. Fortunately, the surface tension prevents yielding after the glass softens. Malecha et al. developed a system with whole PMMA fibers and electronic components embedded inside of the structure [117]. It has immensely improved the system proposed by Golonka et al., providing high sensitivity and low noise level. Authors did not report any problems with the consistency of measurements. Czok et al. manufactured a ceramic absorbance measurement system, where fluorescence was determined indirectly [118]. It contained two vertical glass windows embedded in LTCC, with no reports of integration difficulties or drifting. An entirely new approach has been shown by Malecha by introduction of LTCC bonding with PDMS [119], which—as mentioned earlier—is a very popular microfluidic material. Then, an optical sensor for absorbance measurement has been developed using this technique [120]. The latest method is still in development.

Couceiro et al. [121,122] have developed the monolithic LTCC microsystems for optical measurements. They used 40 µm-thick ceramic tape as the optical window for transmittance measurements. While a transparent window for fluorescence measurements was fabricated by laser ablation of 114 μm-thick LTCC tape. The final thickness after patterning was 20 μm, which was enough to observe light emission. However, the LTCC material exhibit relatively low transmission (below 20%) for the light at wavelengths in the 300–350 nm range. Moreover, the cobalt compounds present in the most popular LTCC material systems (e.g., DuPont 951) absorb light at wavelengths in the range of 525–625 nm [123]. Both of these phenomena limit the application of monolithic LTCC microfluidic systems in absorbance/fluorescence measurements.

## 7. LTCC Technology

The LTCC technology is a very promising alternative for the fabrication of microsystems for analytical chemistry, biology, and medicine. It has a unique feature to integrate electronics and microfluidics in a single module. The manufacturing process of LTCC structures begins with tape casting. From a reservoir containing ceramic-glass powder, mixed with solvent and dispersant, the mixture is transferred onto a support foil and then dried. Thus, the obtained intermediate product is called Green Tape and is very easy to process, usually by milling or laser ablation. Multiple sheets can be laminated in order to develop any 3D structure, just as in additive manufacturing—layer by layer. Moreover, LTCC is foremost a microelectronic material, so integration of electric interconnections is effortless, by use of thick film technology. The last step is firing in ca. 850 °C. More details about the LTCC technology can be found in [124,125].

**Figure 6:**
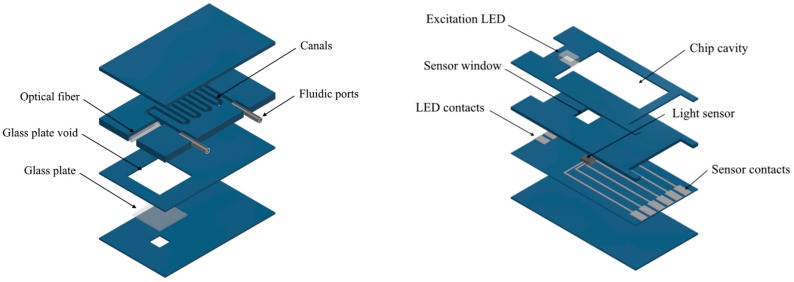
The two-component microfluidic system for fluorescence measurements: the microfluidic chip (**left**) and base station (**right**).

In this work, we want to present the LTCC microfluidic system for fluorescence measurements developed by our group. It consists of a microfluidic chip and base station. Schematic views of both parts are shown in Figure 6. The microfluidic chip is composed of 8 LTCC layers:

- 2 blank at the top,
- 4 with 500 µm wide canals and voids for optical fiber and fluidic ports,
- 1 with a 6 × 6 mm^2^ glass plate and
- 1 at the bottom, with a 2 × 2 mm^2^ window for fluorescence measurement.

The chip was manufactured using commercially available DuPont^®^ DP951 PX LTCC tapes, 254 μm thick. Patterning was carried out using LPKF Protolaser U laser system (λ_max_ = 355 nm). Afterwards, a sodium glass window was inserted in a special void and all layers were stacked together. The lamination process was carried out at a pressure of 10 MPa and a temperature 70 °C for 10 min in an isostatic press. Structures were fired in a box furnace Nabertherm HTC 03/16, using the profile recommended by DuPont^®^ (two step; T_max_ = 875 °C). The glass held well on the smaller window due to surface tension, as described earlier. Afterwards, a PMMA optical waveguide with a diameter of 750 µm and a numerical aperture of NA = 0.5 was glued in. It was then trimmed using a grinding machine and finished with a polishing paper (Thorlabs, Mölndal, Sweden). In order to provide a hermetic fluidic connection, two fluidic ports made of steel capillaries were inserted into the structure.

The microfluidic chip is placed in a base station, which is also manufactured in a LTCC technology. The base station has an indentation for a microfluidic chip for effortless positioning of optical paths and it contains a super bright LED (23N-3, WAH WANG, Hong Kong, China) for analyte excitation, with an emission peak of λ_max_ = 470 nm and a spectral half-width of Δλ_1/2_ = 35 nm, as well as a light sensor (TCS 3414, ams) for fluorescence measurement, with integrated optical filters and an analog to digital converter (ADC). The light sensor provides a signal from 4 canals, which correspond with light filters: clear (from 350 to 650 nm), blue (λ_max_ = 470 nm; Δλ_1/2_ = 110 nm), green (λ_max_ = 524 nm; Δλ_1/2_ = 80 nm), and red (λ_max_ = 640 nm; Δλ_1/2_ = 65 nm). All color filters have a Gaussian spectra. Therefore, the green filter introduces significant selectivity towards fluorescein emission spectra at λ_max_ = 520 nm over LED excitation light.

The presented two-component LTCC-based microfluidic system has been already applied for the fluorescent detection of various chemical and biological agents [80,123]. Baluta et al. constructed a microfluidic FRET-based biosensor for sensitive dopamine detection. The microfluidic detection system was made in the low-temperature co-fired ceramics (LTCC) technology. This sensing system utilized the catalytic oxidation of dopamine to dopamine-o-quinone, and then to poly(dopamine), which can selectively quench the strong luminescence of graphene quantum dots due to Förster Resonance Energy Transfer. The constructed system was used to test pharmacologically labeled dopamine samples with a detection limit of 80 nM [80].

## 8. Conclusions and Discussion

Medical biosensors can diagnose health conditions, such as diabetes, cardiovascular issues, infectious diseases, and cancer. Increasingly, biosensors are also being used to achieve targeted therapies in precision medicine and increase the efficacy of drugs through pharmacogenomics.

The current trend in POCT is inclined strongly towards smart devices equipped with mobile healthcare (mH) [126], which could revolutionize personalized healthcare monitoring and management, thereby paving the way for next-generation POCT [127]. A wide range of mH technologies have already been developed, the most promising being cellphone-based POC technologies for the readout of colorimetric, fluorescent, chemiluminescent, electrochemical, lateral flow, and label-free assays; detection of cells, biomolecules, nanoparticles, and microorganisms; and other diagnostic applications [128,129].

Moreover, over the last two decades, microfluidic technologies (also LTCC) have experienced a significant growth in applications in the field of diagnostics. Microfluidics-based technologies have shown promising applications in several of the main laboratory techniques, including blood chemistries, immunoassays, nucleic-acid amplification tests, and flow cytometry [130]. Microfluidics permits the miniaturization and automation of diagnostic tools for health care applications in POC settings. Due to the idea, Pollock et al. presented the potential of a patterned paper system for monitoring drug-induced hepatoxicity in at-risk individuals (such as those being treated for tuberculosis and/or HIV) [131]. Hugo et al. [132] analyzed the introduction of a centrifugal microfluidic system for POC applications in South Africa (for malaria diagnosis). The use of an automated microfluidic platform for diagnostic of the *Mycobacterium tuberculosis* has also been reported by Jing et al. [133]. Of the all technologies that are used to manufacture microfluidic systems, the LTCC technique seems to be an interesting alternative. It allows easy integration of electronic and microfluidic components on a single ceramic substrate. Moreover, the LTCC material is biologically and chemically inert, and is resistant to high temperature and pressure. The combination of all these features makes the LTCC technology particularly useful for the implementation of fluorescence-based detection in the ceramic microfluidic systems.

However, the realization of new POC diagnostic tools must overcome regulatory defiance. Many occurring microfluidic POC patterns have significant but unproved capability to improve global health. Regulatory procedures, despite being time-consuming, are relevant to assure the effectiveness and reliability of diagnostic tests.

## Figures and Tables

**Table 1:** Fluorescence-based biosensors for hormones determination.

Biomarker	Sensing Platform	Transduction Type	LOD	References
17β-estradiol	Aptamers specific for 17β-estradiol	Evanescent Wave	2.1 nM	[37]
17β-estradiol	anti-E2 antibody	Change in fluorescence intensity	6.37 × 10^−^^6^ ng mL^−^^1^	[38]
17β-estradiol	Fluorescein	Change in fluorescence intensity	30 nM	[36]
Estriol	Graphene oxide/estriol complex	FRET	1.3 nM	[41]
Testosterone	Anti-testosterone antibody	TIRF	0.2 ng L^−1^	[39]
Insulin	Aptamers specific for insulin	FRET	0.72 pM	[40]
Insulin	Anti-insulin antibody	FRET	3.0 × 10^−6^ ng/mol	[34]

**Table 2:** Fluorescence-based biosensors and sensors for neurotransmitters determination.

Biomarker	Sensing Platform	Transduction Type	LOD	References
DA	GQDs/pDA complex	FRET	8 nM	[81]
DA	Functionalized-CuInS_2_ QDs	FI	200 nM	[82]
5HT	APTES-functionalized surface-assembly of Ag@mSiO_2_	Interferometry	84 fM	[83]
5HT	Ehrlich’s reagent-5HT complex	Spectrophotometry	2.3 μM	[84]
DA	dithienotetraphenylsilane/laccase GQDs/pDA complex	FRET	80 nM	[80]
DA	CdSe/ZnS QDs/A	FI	29.3 nM	[85]

**Table 3:** Fluorescence-based biosensors for exemplary metabolites determination.

Biomarker	Sensing Platform	Transduction Type	LOD	References
Lactate	LDH/medical capillary	Change in fluorescence intensity	0.45 mM	[102]
Lactate	LDH/optical fiber	Change in fluorescence intensity	20 μM	[103]
Uric acid	QDs/uricase/HRP	FRET	125 μM	[91]
Uric acid	UCNPs/uricase	IFE	6.7 μM	[101]
Glucose	MIL-53(Fe)/GOx	Change in fluorescence intensity	8.44 × 10^−^^9^ mol L^−^^1^	[104]
Glucose	CdSe/ZnS QDs/concanavalin A	FRET	-	[105]
Glucose	RDC/SNS/GOx	Change in fluorescence intensity	1.2 nM	[106]
